# A Power-Efficient Sensing Approach for Pulse Wave Palpation-Based Heart Rate Measurement

**DOI:** 10.3390/s21227549

**Published:** 2021-11-13

**Authors:** Gabriel Bravo, Jesús M. Silva, Salvador A. Noriega, Erwin A. Martínez, Francisco J. Enríquez, Ernesto Sifuentes

**Affiliations:** Institute of Engineering and Technology, Universidad Autónoma de Ciudad Juárez (UACJ), Ciudad Juárez 32310, Mexico; gbravo@uacj.mx (G.B.); jesilva@uacj.mx (J.M.S.); snoriega@uacj.mx (S.A.N.); emartine@uacj.mx (E.A.M.); fenrique@uacj.mx (F.J.E.)

**Keywords:** wearable health monitoring, resistance-to-time interface circuit, force-sensing resistor, direct microcontroller interface circuit, heart rate measurement

## Abstract

Heart rate (HR) is an essential indicator of health in the human body. It measures the number of times per minute that the heart contracts or beats. An irregular heartbeat can signify a severe health condition, so monitoring heart rate periodically can help prevent heart complications. This paper presents a novel wearable sensing approach for remote HR measurement by a compact resistance-to-microcontroller interface circuit. A heartbeat’s signal can be detected by a Force Sensing Resistor (FSR) attached to the body near large arteries (such as the carotid or radial), which expand their area each time the heart expels blood to the body. Depending on how the sensor interfaces with the subject, the FSR changes its electrical resistance every time a pulse is detected. By placing the FSR in a direct interface circuit, those resistance variations can be measured directly by a microcontroller without using either analog processing stages or an analog-to-digital converter. In this kind of interface, the self-heating of the sensor is avoided, since the FSR does not require any voltage or bias current. The proposed system has a sampling rate of 50 Sa/s, and an effective resolution of 10 bits (200 mΩ), enough for obtaining well-shaped cardiac signals and heart rate estimations in real time by the microcontroller. With this approach, the implementation of wearable systems in health monitoring applications is more feasible.

## 1. Introduction

Health monitoring parameters (such as heart and respiration rate) measured by non-invasive sensing methods have been the object of study over the past decades [1]. In recent years, portable personal monitoring systems or wearable systems have been developed for monitoring the current state of a patient’s health in everyday situations [2]. In the same way, methods for detecting physiological parameters have been designed by implementing monitoring systems in objects of daily use, such as a bed, a toilet seat, a computer mouse, or an electronic weight, to name a few. Furthermore, the integration of the Internet of Things (IoT) into healthcare has led to intelligent applications such as remote healthcare and intelligent healthcare monitoring systems.

Detecting cardiorespiratory signals using electromechanical sensors is a well-known technique used to assess a subject’s health conditions. The mechanical reactions caused by heartbeats and respiration bring about low stress levels, which can be detected by force sensors attached to the body or placed in furniture, such as chairs, mattresses, and automobile security belts [2,3,4,5,6,7,8,9,10]. Formerly, Electromechanical Film (EMFi) and piezoelectric sensors were used; nowadays, Force-Sensing Resistors (FSRs) are preferred because of the simplicity of the interface circuits used to convert the resistance changes into output voltage or current [3]. FSRs are based on piezoresistive sensing technology, which has the advantage of providing an observable resistance change induced by minimal stresses. For this, Polymer Thick-Film FSRs have been implemented in wearable systems for ubiquitous and long-term monitoring, such as sitting posture recognition and muscle contraction, and to detect the cardiac activity from the foot sole [11,12,13,14,15,16].

To measure resistance changes in an FSR, it is common to use analog conditioning circuits that convert these changes into an output voltage. The most common alternative is to place the FSR into a voltage divider or Wheatstone bridge, the current excitation of which depends on the non-actuated resistance of the FSR [9]. In those circuits, the output voltage depends not only on the sensor’s resistance change but also on the excitation voltage or current. Moreover, if the output voltage needed to suit the input range of an analog-to-digital converter (ADC), some analog signal processing stages would need to be implemented, such as amplification, level shifting, and filtering [17]. A more significant number of conditioning stages increases the power consumption and instrumentation size, which restricts the implementation of portable and low-power systems such as wearable wireless sensor nodes for health monitoring.

A simplified circuit to interface resistive sensors has been proposed and widely analyzed [18,19,20,21]. This circuit connects the sensors directly to a microcontroller (MCU) without using an analog signal conditioning path or ADC. In this topology, called Direct Interfaced Circuit (DIC), the MCU excites the resistive sensor to obtain a time-modulated signal digitized by a timer embedded into the MCU. Recently, DIC was used to obtain respiratory signals through a nasal thermistor [22] and by an FSR placed in the seat of a typical chair [16]. This paper presents a novel sensing approach for heart rate monitoring by a DIC (a single FSR connected directly to a low-cost MCU). The main benefits of this approach over traditional skin electrodes and optical sensors are: (1) analog signal conditioning stages can be omitted, which results in a compact and power-efficient HR system; (2) neither analog nor digital filters are needed due to the FSR-yielded, well-shaped, and noiseless heartbeat signal. Hence, HR can be estimated in real time by a simple algorithm implemented in the MCU without any additional digital signal processing. With this sensing approach, wearable systems for ubiquitous health telemonitoring are more feasible.

## 2. Sensing Approach

### 2.1. Heartbeat Signal Detection

**Figure 1:**
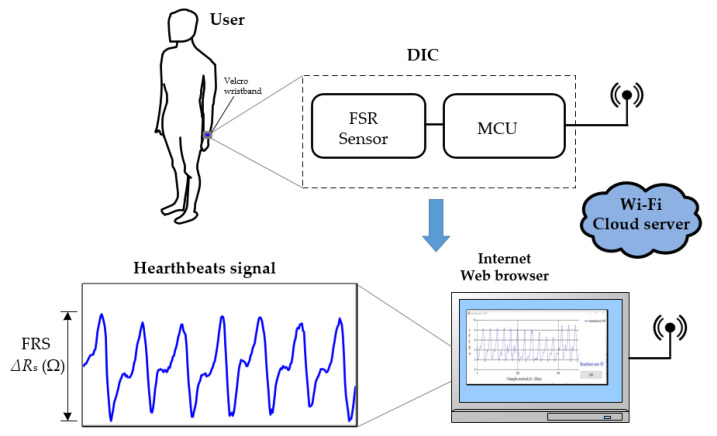
Wearable sensing approach for remote heart rate monitoring.

FSR is a flexible, very low-cost, and robust polymer thick film (PTF) sensor whose resistance (*R*_s_) changes with an increase in the force applied to the sensor’s surface. Heartbeats can be detected by attaching the sensor to the skin in places near the big arteries, which expand their area each time the heart expels blood to the body [23]. Figure 1 depicts the proposed system for sensing the heartbeats of a subject; the FSR is placed near the radial artery with a Velcro wristband and changes its electrical resistance (∆*R*_s_) every time a pulse is detected. Hence, if DIC is able to measure such small-resistance variations caused by cardiac activity, the HR of a subject can be measured. Moreover, the heartbeat’s signal could be transmitted via Wi-Fi to a secured cloud server where any Wi-Fi device could access the report server with proper credential authentication. 

### 2.2. Direct Interface Circuit

**Figure 2:**
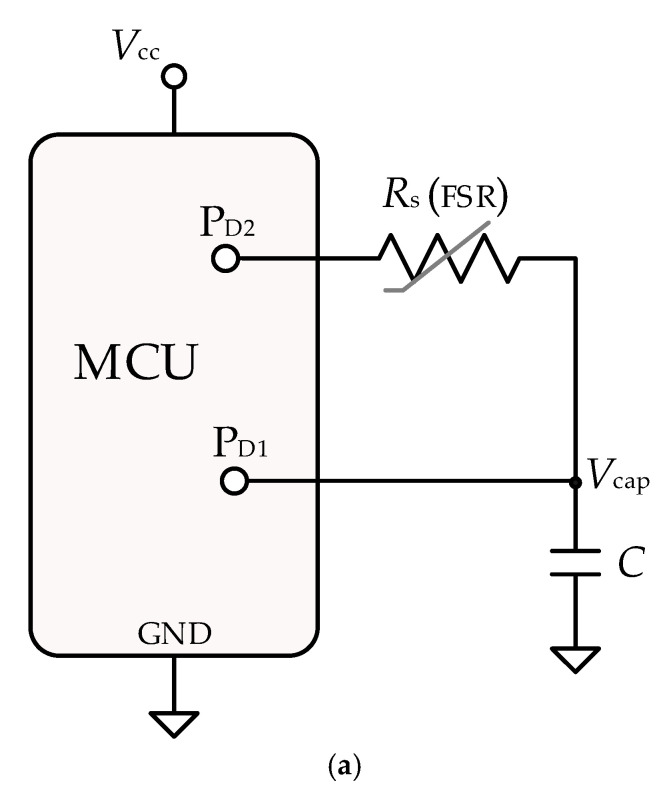

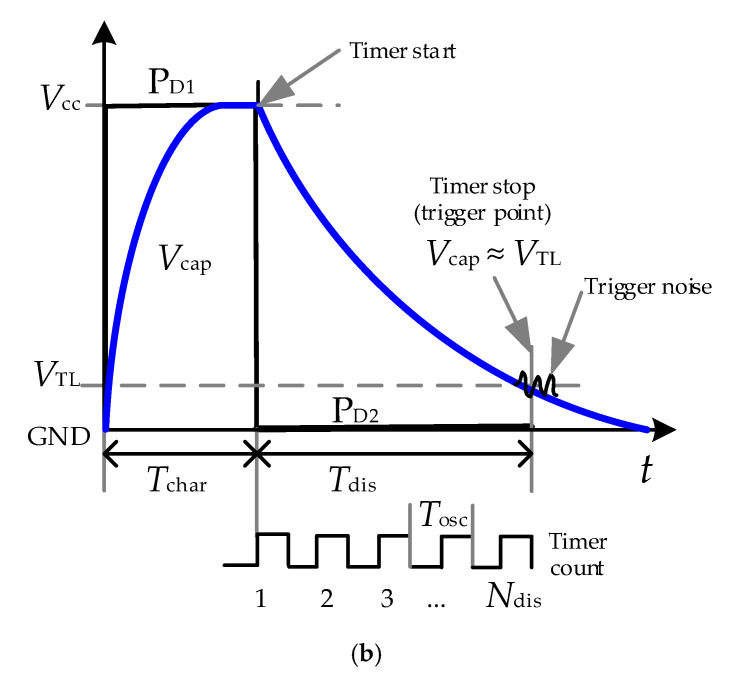
(**a**) DIC to measure the FSR variations (∆*R*_s_) caused by heartbeats; (**b**) digital state of P**_D_**_1_ and P**_D2_** and the voltage across *C* during each measurement of *R***_s_**.

Figure 2a shows the circuit proposed to measure *R*_s_, which is the most basic direct sensor-to-MCU interface circuit [18]. The MCU only needs to have an embedded digital timer and two input/output (I/O) digital pins with external interruption capability. In summary, the DIC simultaneously performs a resistance-to-time conversion and a time-to-digital conversion, resulting in a digital number proportional to *T*_dis_ and hence to *R*_s_. The MCU algorithm to obtain these measurements involves two stages: (a) charging stage and (b) discharging and time measurement stage (see Figure 2b).

In the first stage, P_D1_ is set as an output providing a digital “1”, so P_D1_ generates a step pulse from GND to *V*_cc_ (supply voltage of the MCU) or from “0” to “1” in the digital domain. At the same time, P_D2_ is set as an input, offering a high impedance state “HZ”, so *C* is charged towards *V*_cc_ through *R*_O1_, with a time constant *τ*_c_ = *R*_O1_C. *R*_O1_ represents the internal resistance of P_D1_, whose value is about tens of ohms [18], small enough to achieve a fast charging time. The charging stage must be at least *T*_char_ = 5*τ*_c_ to ensure that *V*_cap_(*t*) reaches *V*_cc_. In the second stage, P_D2_ is set as an output providing a digital “0”; consequently, *C* is discharged towards GND through *R*_x_, with a time constant *τ*_d_ = *R*_s_*C*, while the embedded timer of the MCU starts to measure the time interval required to do so. P_D1_ is configured as an input to interrupt the MCU on the falling edge. When *V*_cap_ ≈ *V*_TL_ (low threshold voltage of the Schmitt Trigger (ST) buffer embedded into P_D1_), the ST triggers and the timer stops. The count of the timer *N*_dis_ is the digital equivalent to the discharging time, *T*_dis_, proportional to *R*_s_ as:

(1) $$T_{\mathrm{dis}}=N_{\mathrm{dis}}T_{\mathrm{osc}}=R_{s}C\;\ln\left(\frac{V_{\mathrm{CC}\;}}{V_{\mathrm{TL}}}\right)$$

where *T*_osc_ is the period of the clock signal of the timer. *R*_s_ can be estimated from (1), assuming that *C*, *V*_cc_, and *V*_TL_ are known. The variability of these parameters can be compensated for by adding reference components and then applying auto-calibration techniques [18,19,20]. In this application, the DIC (Figure 2a) does not require any reference resistor because the information to be monitored (cardiac pulse signal) is contained in the change in *R*_s_, not in its absolute value, so the circuit’s accuracy is less critical. On the other hand, the slew rate, *SR*, of the exponential discharging waveform at the trigger point is:

(2) $$SR=\frac{V_{\mathrm{TL}}}{R_{s}C}$$

Ideally, the time-to-digital conversion has a resolution equal to *T*_osc_. However, the main uncertainty sources are the quantization of the timer and the trigger noise (the point when *V*_cap_ ≈ *V*_TL_ and the timer stops is a noise-sensitive process) [24]. If only quantization effects are considered, the resolution of the DIC in bits, *M*, or ohms, *r*, respectively, is:

(3) $$M=\mathrm{lb}\left(\frac{T_{\mathrm{dis},\max}-T_{\mathrm{dis},\min}}{T_{\mathrm{osc}}}\right)$$

(4) $$r=\frac{R_{s,\max}-R_{s,\min}}{2^{M}}$$

where *R*_s,min_ and *R*_s,max_ are the minimal and maximal resistance values that produce, respectively, the discharging times to be measured: *T*_dis,min_ and *T*_dis,max_.

From (1) and (3), a high resolution can be achieved by decreasing *T*_osc_ or increasing *C* (thus *τ*_d_). However, a high value of *C* increases the measuring time, thus reducing the bandwidth of the measurement system. So, a *C* value that yields an optimal resolution must be selected [25]. By replacing (1) in (3) for a particular application requiring *M* bits of resolution, the *C* value should be:

(5) $$C\geq \frac{T_{\mathrm{osc}}2^{M}}{\left(R_{s,\max}-R_{s,\min}\right)\ln\left(\frac{V_{\mathrm{CC}\;}}{V_{\mathrm{TL}}}\right)}$$

On the other hand, the trigger noise superimposed on the exponential discharging voltage on *V*_TL_ limits the resolution of the measurement system to an effective number of bits (ENOB) lower than *M*. According to [24], the ENOB can be estimated by:

(6) $$\mathrm{ENOB}\approx M-\mathrm{lb}\left(\frac{\sqrt{u^{2}\left(T_{\mathrm{dis},\max}\right)+u^{2}\left(z\right)}}{u\left(z\right)}\right)$$

where *u*(*T*_dis,max_) is the standard uncertainty of *T*_dis,max_ due to noise effects and *u*(*z*) represents the quantization uncertainty that can be estimated as the standard deviation of the uniform distribution of width *T*_osc_, that is:

(7) $$u\left(z\right)=\frac{T_{\mathrm{osc}}}{\sqrt{12}}$$

The variability of *T*_dis_ can be analyzed by a statistical analysis of a series of observations. The histogram, the mean, and the standard deviation, *s*(*T*_dis,max_), permit us to characterize noise effects. So, *u*(*T*_dis,max_) from *k* observations can be estimated by:

(8) $$u\left(T_{\mathrm{dis},\max}\right)=\frac{s\left(T_{\mathrm{dis}}\right)}{\sqrt{k}}$$

In summary, from (2), a small *R*_s_*C* yields large *SR*, so quantization effects predominate over trigger noise, and the ENOB is equal to *M*. Conversely, a large *R*_s_*C* implies slow *SR*, which makes the triggering process more susceptible to noise, increasing the count dispersion and the standard deviation *s*(*T*_dis_). So, there is a speed/resolution trade-off that depends mainly on *R*_s_*C*. By establishing a measurement range, *R*_s,max_–*R*_s,min_, in a particular application to achieve *M* bits of resolution, equation (5) can be used as a first design rule to estimate *C*.

## 3. Materials and Method

**Figure 3:**
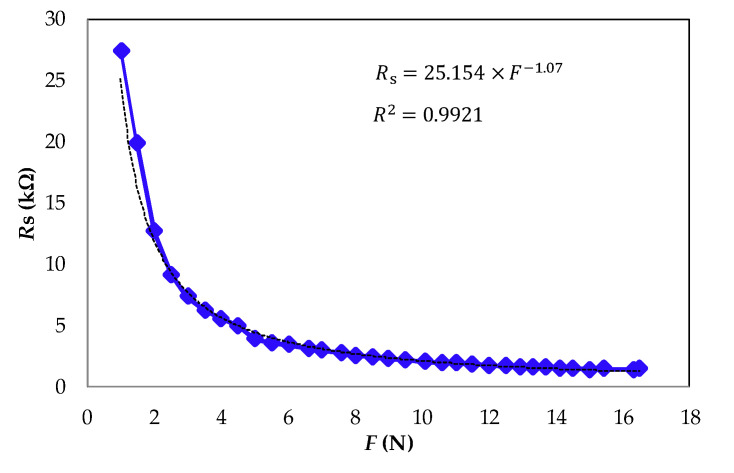
Experimental response of the FSR-402 when force is applied to the sensor’s surface.

The DIC (Figure 2a) was implemented by a D1 mini ESP32 module. This module was designed for mobile electronics and Internet-of-Things (IoT) applications. It has an MCU (ESP32 from Espressif Systems) that contains Wi-Fi and Bluetooth (BLE embedded) and 520 kB of RAM. The module was powered by a rechargeable battery 5 V at 1 A and 2.2 Ah, and the MCU with *V*_cc_ = 3.3 V by a voltage regulator incorporated in the module. *R*_s_ was an FSR-402 sensor (from Interlink Electronics) with a sensible area of 14.68 mm in diameter, a nominal thickness of 0.46 mm, *R*_s_ > 10 MΩ without force applied, and rise time of <3 µs. To know the sensor response, known forces from 1 N to 16.5 N were applied (Figure 3). In addition, the nominal *R*_s_ value was measured with a digital multimeter when the FSR was placed in a Velcro wristband near the radial artery, the nominal value was* R*_s_ = *R*_s,0_ *≈* 5 kΩ.

The MCU program to measure *T*_dis_ (and so *R*_s_) by following the sequence described in Section 2.2 was implemented in C language. P_D1_ and P_D2_ (Figure 2a) were implemented by GPIOs 13 and 14, respectively. (The MCU works at 80 MHz, so the digital timer measures *T*_dis_ with a resolution of *T*_osc_ = 12.5 ns.) From each measurement of *T*_dis_, *R*_s_ was obtained from (1). The MCU parameters *V*_cc_ = 3.23 V*, V*_TL_ = 1.56 V, *R*_O1_ = 32 Ω were obtained from the procedures described in [18]. By considering ∆*R*_s,max_ *= R*_s,max_ − *R*_s,min_ ≈ 200 Ω and *M =* 10 bits, from (5), the minimal value of *C* is 86 nF, a metallized polypropylene with *C =* 150 nF was chosen. According to [25], a DIC with these values would be capable of a maximum sampling rate of 1.75 kSa/s, more than enough for this application.

**Figure 4:**
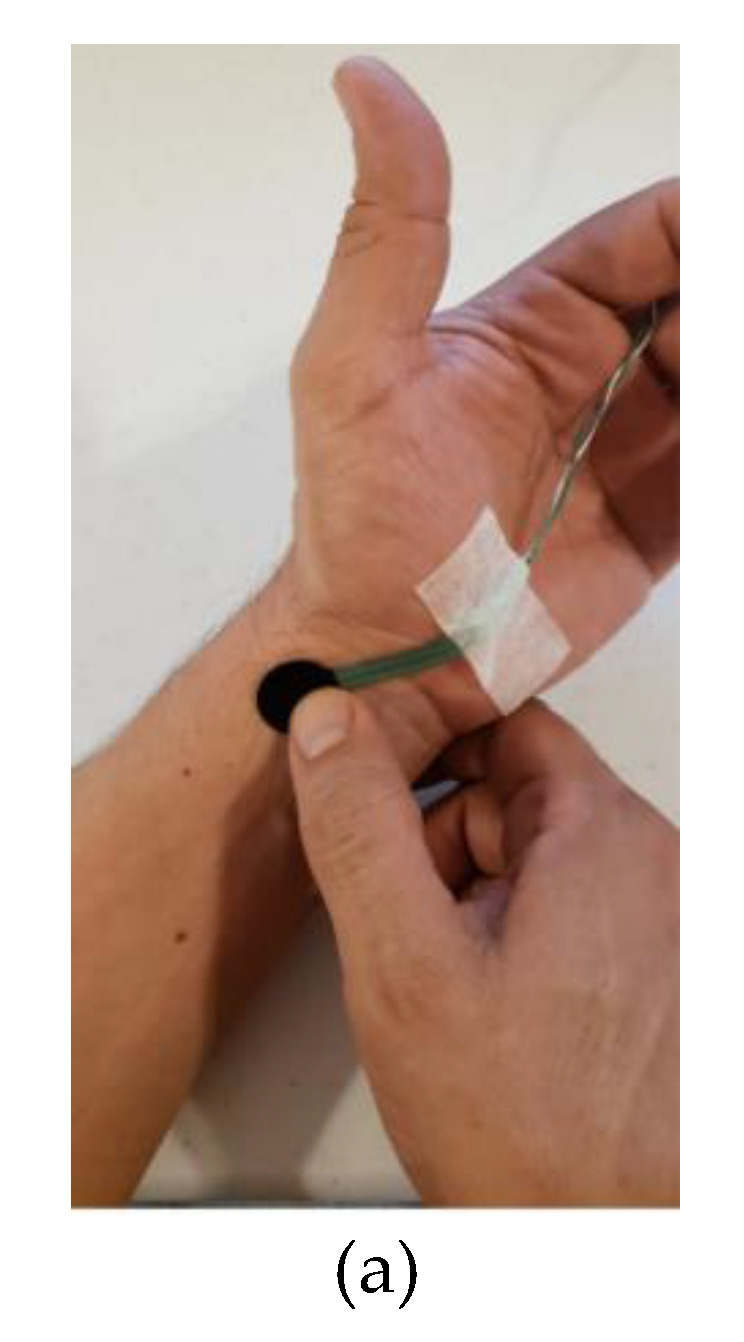

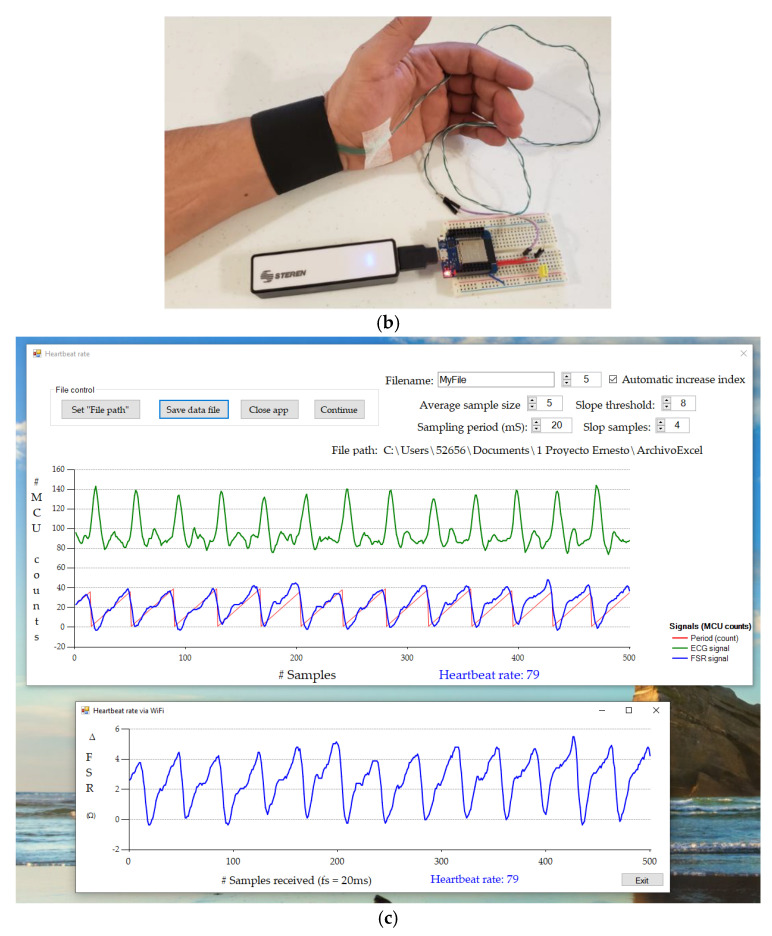
Prototype of the wearable sensing approach for remote heart rate monitoring. (**a**) FSR placed over the radial artery; (**b**) FSR with a Velcro wristband connected to a DIC; (**c**) graphical interface for remote visualization of the heartbeat signal and HR value.

An adult’s regular resting HR ranges from 60–100 BPM, i.e., between approximately 1 and 2 beats per second. For a reasonable reconstruction of the heartbeat signal, a sampling rate of *S_f_* = 50 Sa/s was selected, which was configured and controlled by an internal digital timer. Hence, the DIC takes a sample of *R*_s_ every 1/*S_f_ =* 20 ms and stores it in its RAM. The acquisition time for *N*_s_ samples was defined as *T*_acq_ *= N*_s_ (1/*S_f_*). For concurrent records of 10 s (of heartbeat signal), the MCU was programmed to measure and save (in RAM) 500 samples of *R*_s_. Simultaneously, the MCU calculates the heart rate from the samples in real time by taking the period from two consecutive slopes, based on the abrupt downward slope shown in each beat of the signal (see Figure 4c). Each record of 500 samples and heart rate value was sent via Wi-Fi to a secured cloud server.

The sensing approach for remote heart rate monitoring was experimentally tested with five volunteers. Volunteers were asked to remain quiet during the measurement (10 s approximately) to avoid motion artifacts caused by hand movements. The FSR was attached with a Velcro band directly on the skin near the radial artery, as shown in Figure 4b. A commercial heart rate monitor (pulse sensor from World Famous Electronics) was used as a comparison to test the feasibility of the proposed system. This device combines a simple optical heart rate sensor with amplification and noise cancellation circuitry, making it fast and easy to obtain reliable pulse readings. Both signals were acquired by the same MCU: the FSR signal was acquired by the DIC interface algorithm (blue curve in Figure 4c), and the signals from the commercial monitor by the embedded ADC (green curve in Figure 4c). The graphical visualization of both signals was realized in a PC program implemented in Visual C#^TM^. The communication between the PC and the prototype for this test was via Wi-Fi and USB.

We used the Bland–Altman plot to examine the agreement between the proposed wearable system and commercial heart rate monitor. Ten records of heartbeat signals were taken from each volunteer. As proposed in [26,27], the Limit of Agreement (LoA) was obtained with a 95 % confidence interval as [*µ* − 1.96*σ*, *µ* + 1.96*σ*], where *µ* is the average difference, and *σ* is the standard deviation. Pearson’s correlation between the two measurement systems was also estimated.

## 4. Experimental Results and Discussion

Figure 3 shows the experimental response of the FSR-402 obtained from the characterization. It shows how the resistance (*R*_s_) decreases nonlinearly as force is applied to the sensor’s surface. Nonlinearity errors could modify the shape of the heartbeat signal but not the HR estimation. Figure 4 depicts the wearable sensing approach for remote heart rate monitoring: (a) FSR-402 placed directly on the skin, (b) FSR with a Velcro wristband near the radial artery connected to a DIC, and (c) remote visualization of the heartbeats signal and HR value.

**Figure 5:**
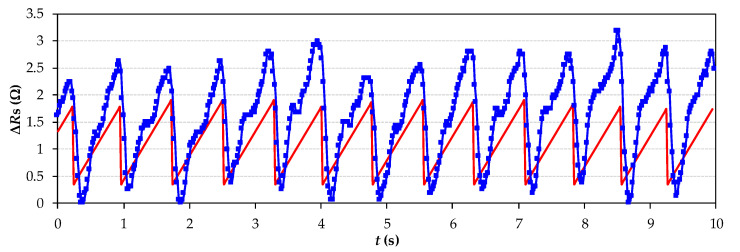
Experimental heartbeat signal reconstruction and HR measurement by the downward slope shown in each signal beat (the MCU implements the algorithm in real time).

Figure 5 shows how the MCU reconstructs the heartbeat signal by a sampling rate of 50 kSa/s and 500 samples (a record of 10 s). Simultaneously, the MCU calculates the HR by taking the period from two consecutive abrupt slopes, beat per beat of the signal (Sawtooth signal). In this case, HR was 79 BPM (see Figure 4c).

**Figure 6:**
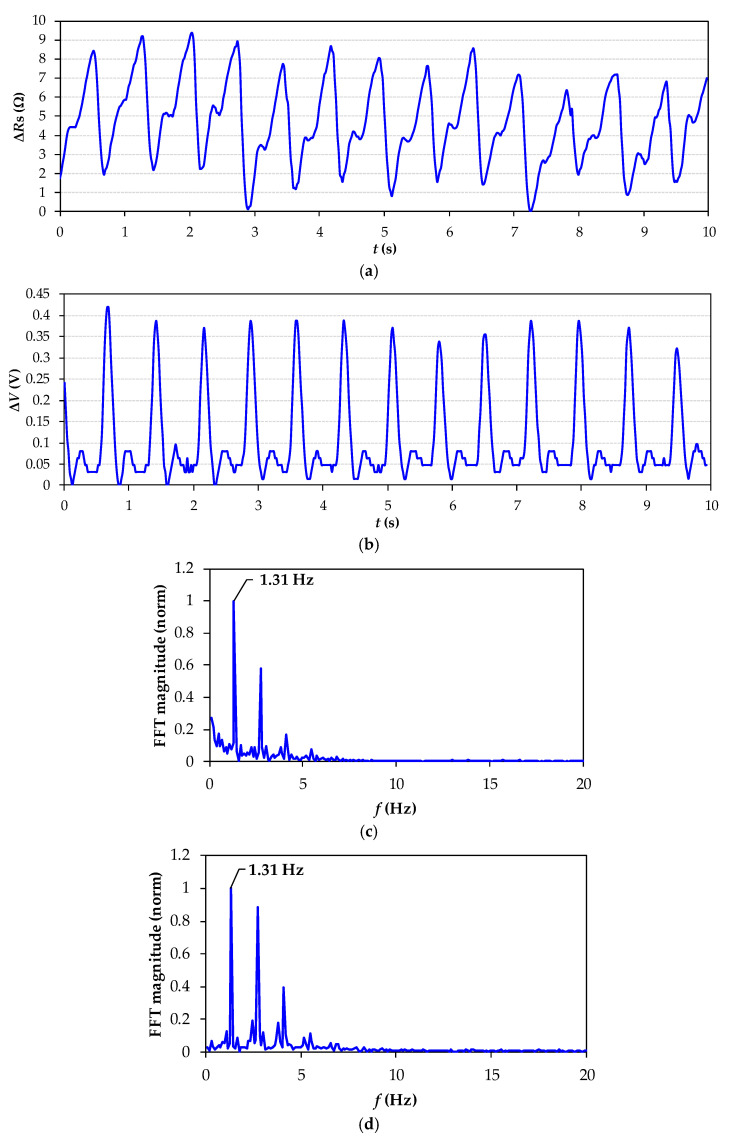
Experimental heartbeat signal from S1. (**a**) Resistance variations in FSR measured by the DIC when the sensor was located near the radial artery; (**b**) voltage variations in the commercial heart rate monitor; (**c**) frequency spectrum of the FSR signal; (**d**) frequency spectrum of the commercial monitor signal.

**Figure 7:**
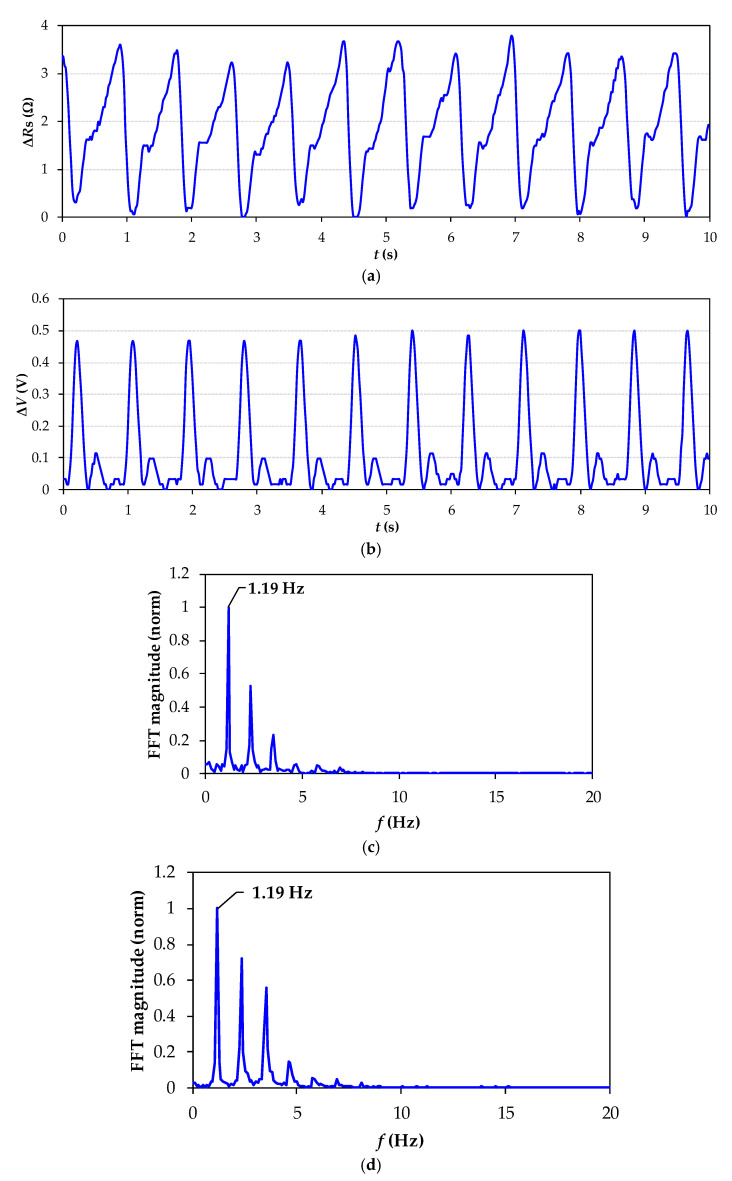
Experimental heartbeat signal from S2. (**a**) Resistance variations in FSR measured by the DIC when the sensor was located near the radial artery; (**b**) voltage variations in the commercial heart rate monitor; (**c**) frequency spectrum of the FSR signal; (**d**) frequency spectrum of the commercial monitor signal.

Figure 6a and Figure 7a show the resistance variations ∆*R*_s_ of the FSR measured by the DIC (Figure 2a) of volunteers S1 and S2, respectively. In the same way, Figure 6b and Figure 7b show the voltage variations, ∆*V*, of the commercial HR monitor. Comparing the signals, a peak resistance change on *R*_s_ occurs after a QRS complex, implying that the signal registered with the DIC is related to cardiac activity. The heartbeat signal obtained from the FSR system is consistently compared with that obtained from the commercial monitor. In all cases, both signals match the number of beats. The FSR changes its electrical resistance each time a pulse is detected, and this pulse rate refers to the contraction and expansion of the artery when blood passes. Each time the heart expels blood into the body, the radial artery expands its area, applying pressure on the FSR and causing a downward change in the resistance of the FSR. Consequently, because the FSR is a sensor with a negative force coefficient, the shape obtained by the FSR corresponds to an inverted cardiac pulse signal. Since this is the natural response of the FSR sensor and does not affect the heart rate estimation, nothing further was performed with this result. As a result, the DIC was able to detect resistance changes caused by cardiac activity, enough to obtain well-shaped heartbeat signals and HR estimation. These were small-signal resistance variations of around ±3.5 Ω (for S1) and ±1.5 Ω (for S2). On the other hand, the sensitivity of the proposed method relies on the sensor sensitivity. Since the FSR is not supplied by any constant voltage or current, self-heating problems are avoided, and the sensitivity does not depend on any polarization source, as usually happens in conventional signal conditioning systems.

Figure 6c,d show the frequency spectrum of the heartbeat signal for S1; in both figures, there was a fundamental peak at 1.31 Hz (79 BPM). In the same way, Figure 7c,d show the frequency spectrum of the heartbeat signals for S2; in both cases, there was a fundamental peak at 1.19 Hz (72 BPM). These results demonstrate that the proposed wearable system is able to detect cardiac activity beat by beat, and it is also able to detect different heart rates. Furthermore, the FSR signals have a negligible contribution of noise, which facilitates the estimation of HR.

**Figure 8:**
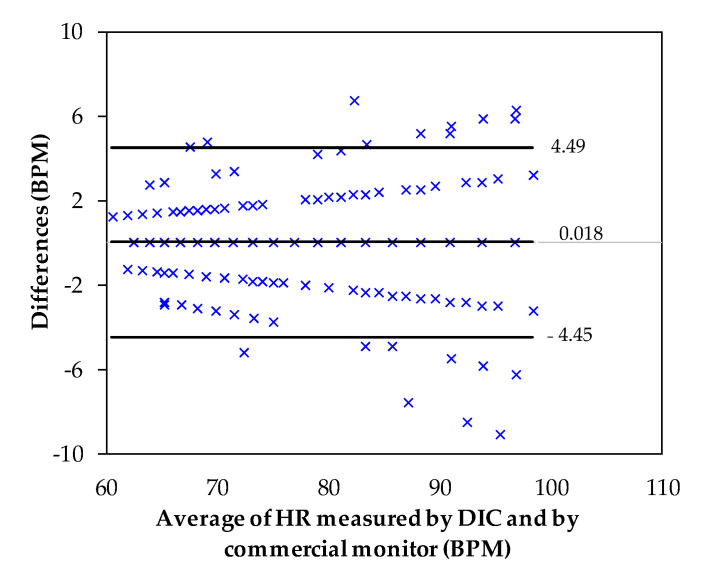
Bland–Altman plot that compares the HR measured by DIC and by the commercial monitor peak to peak.

**Figure 9:**
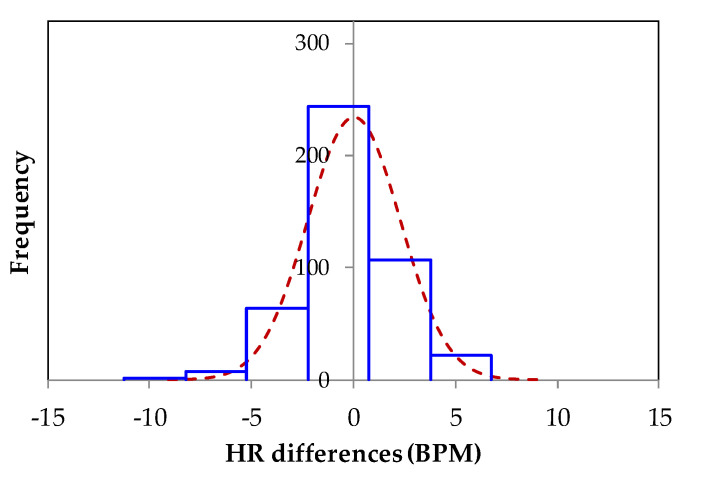
Histogram of the peak-to-peak differences between the HR measured by the DIC and by the commercial monitor.

**Figure 10:**
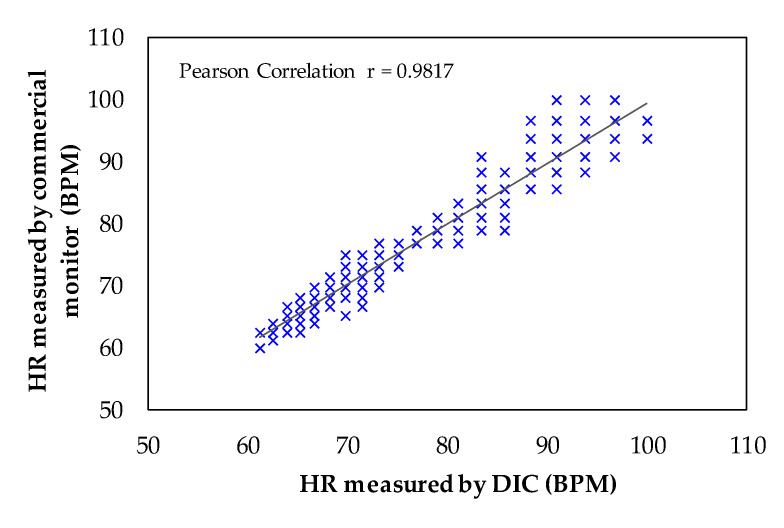
Scatter plot of the peak-to-peak HR measured by the DIC and by the commercial monitor.

The Bland–Altman plot that compares the heartbeat signals (peak to peak) measured from the five volunteers (500 records) by the DIC and by the commercial monitor is shown in Figure 8. The average difference, the standard deviation, and LoA were, respectively: *µ* = 0.018, *σ* = 2.28, and [−4.45, 4.49] all in BPM. The histogram in Figure 9 shows a normal distribution of the peak-to-peak differences from the HR measured by DIC and by commercial monitor. As observed in the Bland–Altman plot, from 60 BPM to approximately 96 BPM there is a high agreement between the two measurement systems. The scatter plot between the HR measured by the DIC and by commercial monitor is given in Figure 10. The Pearson coefficient was 0.9817, which is a statistically significant correlation. Therefore, it is shown that there is an excellent coincidence between the signals obtained with the DIC and the commercial system.

A summary of wrist-type PPG methods for HR monitoring is presented in Tables 4 and 5 from [28]. To compare our proposed sensing approach for HR measurement, we selected those techniques whose performance was evaluated by Bland–Altman analysis and Pearson’s coefficient. The experimental performance of our system (LoA: [−4.45, 4.49] BPM, *σ* = 2.28 BPM, and Pearson coefficient of 0.9817) yielded similar results to six of the eleven methods presented in Tables 4 and 5 from [28].

As is well known, the presence of motion artifacts (MA) in the analysis and interpretation of cardiovascular signals, obtained by different methods ranging from mechanical to electrical and optical systems, is inevitable [29]. This work aims to estimate the HR of subjects in a quasi-stationary position avoiding MA caused by hand movement during the measurement (10 s approximately). As shown in Figure 6 and Figure 7, the FSR signals measured by the DIC when the sensor was located near the radial artery have a negligible noise contribution, which facilitates the estimation of HR. The experimental results prove that it is possible to estimate the HR in real time using a DIC without any additional digital signal processing.

## 5. Conclusions

A force-sensing resistor, such as an FSR-402, placed in a wristband near the radial artery and connected directly to a low-cost MCU yields a reliable, compact, and low-cost heart rate monitor. The experimental results show that small-resistance variations caused by the heartbeats of a subject bring well-shaped heartbeat signals from which it is possible to estimate the HR in real time by a simple MCU algorithm without any additional digital signal processing. Using this approach, wearable systems or wireless sensor nodes for health monitoring with high power efficiency are feasible.

## Data Availability

Not applicable.
